# Sex, age, and family structure influence dispersal behaviour after a forced migration

**DOI:** 10.1017/ehs.2023.16

**Published:** 2023-06-08

**Authors:** Jenni J. Kauppi, Simon N. Chapman, Jenni E. Pettay, Mirkka Lahdenperä, Virpi Lummaa, John Loehr

**Affiliations:** 1 Department of Biology, University of Turku, 20014 Turku, Finland; 2 INVEST Flagship Research Centre, University of Turku, 20014 Turku, Finland; 3 University of Helsinki, 00014 Helsinki, Finland

**Keywords:** Migration, evacuation, individual characteristics, social group

## Abstract

Dispersal does not only mean moving from one environment to another, but can also refer to shifting from one social group to another. Individual characteristics such as sex, age and family structure might influence an individual's propensity to disperse. In this study, we use a unique dataset of an evacuated World War II Finnish population, to test how sex, age, number of siblings and birth order influence an individual's dispersal away from their own social group at a time when society was rapidly changing. We found that young women dispersed more than young men, but the difference decreased with age. This suggests that young men might benefit more from staying near a familiar social group, whereas young women could benefit more from moving elsewhere to find work or spouses. We also found that having more younger brothers increased the propensity for firstborns to disperse more than for laterborns, indicating that younger brothers might pressure firstborn individuals into leaving. However, sisters did not have the same effect as brothers. Overall, the results show that individual characteristics are important in understanding dispersal behaviour, but environmental properties such as social structure and the period of flux after World War II might upend the standard predictions concerning residence and dispersal.

**Social media summary:** Individual characteristics influence dispersal away from social group after a forced migration in a Finnish population

## Graphical abstract

**Figure:**
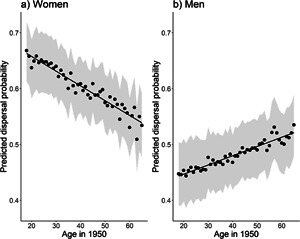

## Introduction

Natal dispersal is the movement of an individual away from its point of origin to a new social group or settlement habitat where he or she usually reproduces (Clobert et al., 2001; Greenwood, 1980). It has consequences on individual fitness, population dynamics and genetic structures, as well as distributions of species (Bonte et al., 2012; Bowler & Benton, 2005; Clobert et al., 2001; Greenwood, 1980), and it is the main reason for gene flow between populations (Greenwood, 1980). Dispersal strategies evolve in response to the benefits, such as more resources, better habitat or mating opportunities, and costs, such as increased mortality or loss of social ties, associated with moving to new locations (Bowler & Benton, 2005; Clobert et al., 2012; Creel & Creel, 2015). In social species, groups of relatives might even disperse together in coalitions to escape unfavourable conditions (Koenig et al., 2000; OECD, 2017; Packer & Pusey, 1993; Sharp et al., 2008).

Among humans, a social group usually entails the members being in some sort of interrelation, sharing common characteristics such as interest, values, ethnic or social background, or kinship ties (e.g. marriage, common ancestry, adoption; Macionis & Gerber, 2010; OECD, 2017; Reicher, 1982). Studies of human dispersal – or migration in some cases – are therefore not only crucial to understanding behaviour from an evolutionary point of view but also relevant for many contemporary world challenges. Large-scale migrations happen for numerous economic, environmental, social or political reasons such as wars and other conflicts, environmental events and natural disasters (e.g. floods, droughts, sea level rise) (Science for Environment Policy, 2015), and various studies from different perspectives are needed to understand the causes and effects of these movements. As humans inhabit an exceptionally wide range of social and ecological environments (Clarke & Low, 1992), it is reasonable to assume that the proximate reasons for dispersal change throughout life history and that dispersal is very context dependent and may be influenced by multiple factors together (d’Errico et al., 2012).

Dispersal patterns are not universal: they can differ over time, and vary between societies, cultures and contexts (Lynch et al., 2022; Marlowe, 2004; Moravec et al., 2018). Previous studies have focused on the influence of individual characteristics and the social group affecting dispersal decisions in humans, but more research is needed to know how individuals disperse based on features such as their sex, age and family relations in specific social contexts and situations. First, dispersal is often sex biased in mammals with males generally dispersing more frequently and farther than females (Greenwood, 1980). The differences in dispersal behaviour between men and women stem from the benefits and costs they face from their society and culture (Chen et al., 2023; Scelza, 2011). These include, for example, which sex benefits more from staying and which more from leaving in terms of pressures such as resources, work, kinship or marriage (Towner, 2002; Wood et al., 1985). A previous study of human dispersal on a nineteenth-century Swedish population found that women had a higher probability of dispersal and men were more philopatric, argued to be due to resource competition for men and reproductive advance for women (Clarke & Low, 1992). Similarly, the proportion of dispersing individuals in the United States and northwest Germany in eighteenth and nineteenth centuries was somewhat higher among women than men (Beise & Voland, 2008; Towner, 2001). Accordingly, women are more influenced by marriage-related dispersal, and in patrilocal societies, post-marital residence patterns drive women to live near their spouse's family rather than their own (Marlowe, 2004; Towner, 2002). However, as humans are very fluid in their dispersal patterns, there are societies in which women remain more in their natal locality (especially in matrilocal communities) and men move more (Koster et al., 2019).

Second, different constraints or pressures to disperse may exist at different ages, and therefore the propensity to disperse differs as well (Bowler & Benton, 2005). In many mammals, dispersal occurs shortly after behavioural independence from the parents at specific ages (Clobert et al., 2012). For example, human dispersal peaked at ages between 20 and 24 years in the nineteenth century Sweden (Clarke & Low, 1992). Pressure to establish one's own territory might drive individuals to disperse at a young age (Mayer et al., 2017), but alternatively, an individual might disperse at older ages if waiting could prove beneficial. In contemporary Europe, it is most common for young individuals to leave their parental home for work, education or social advancements (marriage, relationship) between the ages of 20 and 30 years (Angelini et al., 2011), but such age effects or their drivers might also differ between genders; in patrilocal societies dispersing at a young age for marriage is more common practice among women than in men (Clarke & Low, 1992; Marlowe, 2004; Towner, 2002). In contrast, young men might be more motivated to disperse owing to, e.g., better work environment or better opportunity for resources.

Finally, the presence of parents and other kin such as siblings is an important component affecting dispersal patterns, particularly in social species (Clobert et al., 2012). Kin interactions can be both beneficial or costly; dispersal can decrease kin competition for limited resources and mates, but staying with kin can provide benefits to an individual through cooperation with relatives (Lambin et al., 2001). In humans, inheritance practices, wealth and social standing of an individual's parents may shape dispersal patterns (Towner, 2001), but also the parental investment and family social structure can cause variation in dispersal behaviour among children (Clarke & Low, 1992; Towner, 2001; Voland & Dunbar, 1997). According to Clarke and Low (1992), birth order influences dispersal among men in nineteenth-century Sweden, since the youngest of a large family had the smallest likelihood of inheriting resources and therefore were most likely to disperse in comparison with those born earlier or to smaller families. Nitsch et al. (2016) showed that historical Finns had an increased probability of dispersal when same-sex elder siblings resided in their natal area: inheriting land led firstborn sons of land-owning families to be more philopatric than their younger siblings, whereas female dispersal increased with the number of elder sisters regardless of their social status. Similarly, in historical Germany, having more brothers increased the odds of dispersal for sons of farmers compared with those with fewer than two brothers, but not for sons of workers, whereas two or more sisters increased the dispersal probability of daughters of workers, but not that of daughters of farmers (Beise & Voland, 2008). Thus, in humans, sibling interactions seem to be an important factor driving an individual's decision to disperse, at least in certain situations and societies. Consequently, sex bias also shapes the kinship and social group ties the individual will have in their lifetime. Depending on which sex is more philopatric, the philopatric individuals will have more closely related kin living closer than the dispersing individuals, although having fewer relatives nearby does not necessarily mean that they have less help or support available when needed (Power & Ready, 2019).

In this study, we test how individual characteristics such as sex, age, number of siblings and being firstborn or laterborn influence an individual's subsequent dispersal away from their own in-group in a unique context following a forced migration. To test this, we use a well-documented dataset of recorded movements and life histories of a Finnish population which was evacuated from the region of Karelia during World War II owing to loss of territory and relocated elsewhere in Finland. Some relocated individually, and others with the resettlement of entire evacuated villages to new areas, thus maintaining old social bonds. This is because during the evacuation process, the Finnish government created placement plans in order to help the Karelian evacuees to find new permanent residency in Finland. The placement plans had a goal to place the farmers in areas similar to their old living areas in natural conditions, transport and communications, and economic conditions, as well as to keep old neighbourhood relationships intact to preserve social networks, and keep old Karelian municipalities united in culture and language (more in Materials and methods). After the evacuation and re-settlement, individuals were free to move away from the assigned locations if they so preferred. This provides an opportunity for us to test the influence of the individual characteristics on dispersal away from their in-group. In-group is defined here as an individual's birth municipality community before the war.

Since the life histories, movements and occupations of evacuated Karelians were extremely well recorded after the war, these data provide a unique opportunity for us to study human dispersal behaviour away from their familiar social environment. In particular, the dataset provides a quasi-natural experiment to study dispersal motivations not usually available in humans. Here, the choice of dispersal or relocation is not the product of previous individual conditions that may affect health, sociality and fitness, but determined by war events and government decisions. The quasi-natural experiment also circumvents problems with heterogeneity in potential trauma before the relocation that could interact with integration behaviour after settlement and confound results, because everyone was exposed to the same trauma, as they were forced to leave Karelia regardless of any desire to migrate.

The study period allows predictions to be tested concerning residency and dispersal behaviour during the period of flux after World War II when the traditional society of Finland changed considerably, transforming from an agricultural to a non-agricultural and more modernised society (Sarvimäki & Jäntti, 2019), and experiencing a rapid growth of the industrial production and economy (Statistic Finland, 2007). Although dispersal patterns have been well studied in humans, especially the differences between men and women, our study provides new insights into dispersal patterns during a time of societal change (i.e. industrialisation, urbanisation, mobility in social status). Also, women's status in the society gradually improved from the early twentieth century onwards; for example, women got better education rights, the right to vote and the right to own property independently from their husbands. These changes had impact on agricultural traditions as well. Traditionally (at least until late nineteenth century Finland) sons, especially firstborn sons, were usually favoured and they were the first to inherit farm and land from parents. However, sometimes all sons got an equal share of capital, cattle or personal property and daughters got half a share, and if daughters inherited the farm their husband became the head of the farm at marriage. If the firstborns inherited most of the property, then they were obligated to pay their siblings their due, which was lower in value in comparison with the value of the farm and land (Faurie et al., 2009; Moring, 1993, 2003; Silvasti, 2010). It is important to notice that as the society changed these inheritance practices also slowly started to shift to more modern practices (more equal shares in inheritance). However, even if at the time of the study these inheritance practices were not followed as strictly and children inherited resources more equally than described, the patriarchal history of the society might still have influenced individual behaviour, and the dispersal decisions of Karelian farmers, especially based on their sex and birth order.

We predict that dispersal away from the social group after the war is more common among women than among men, but both sexes have both stayers and leavers similar to findings of previous studies (Clarke & Low, 1992; Towner, 2002; Nitsch et al., 2016). Women might be more inclined to leave their familiar social group because of, e.g., marriage opportunities, even though we are unable to use marriage as a prediction variable owing to a lack of available data on marriage histories. Men on the other hand could be more motivated to stay if their social group provides more support in farming and better resources. We predict dispersal to be most common for individuals between the ages of 20 and 30 years for both sexes (similar to the findings of Clarke & Low, 1992), and because it is most common for young adults to move away from home and their parents (Angelini et al., 2011). Brothers and sisters are predicted to increase the propensity of individual dispersal in order to reduce competition for parental resources, especially for young rural individuals with multiple same-sex elder siblings. Firstborn individuals might be less keen to disperse, i.e. they should stay more with their in-group, since they might be the first in order to inherit parental resources, and they also might have most pressure to stay and help the family.

## Materials and methods

### Study population and historic background of the data

During World War II, Finland lost a portion of Karelia to the Soviet Union and 420,000 Finns needed to be evacuated and relocated from occupied Karelia into non-occupied Finland. It was very important for the evacuees to record the history and memories of Karelians into journals, and therefore lives, movements, memories and histories of Karelian evacuees were collected into a book series called ‘Siirtokarjalaisten tie’ (Anon.) published in 1970. These records have subsequently created an excellent basis for research from multiple different perspectives (Loehr et al., 2017; Lynch et al., 2019a, 2019b, 2020; Pettay et al., 2021).

In November 1939, the Winter War started when the Soviet Union invaded Finland, and evacuation from Karelia was necessary (11% of the Finnish population). It was thought that this was only a temporary military procedure and that people would soon be able to return back to Karelia. However, the Moscow Peace Treaty, signed by Finland and Soviet Union in 1940, forced Finland to cede areas of Karelia to the Soviet Union. Everyone from these Karelian areas were evacuated to the west, and over 420,000 Karelians lost their homes. The evacuated people were moved to designated placement areas, with each municipality of Karelia assigned its own placement municipality elsewhere in Finland. These plans were not well executed, and multiple people were moved several times to new places by the Finnish government, until the start of the Continuation War in June 1941. Finland regained the territories of Karelia lost in the Winter War, allowing Karelians to return to their home areas already in 1941, and by the spring of 1944 over 65% of Karelians had returned. When the overall war situation started to worsen for Finland in 1944, the second evacuation plan started to take place. In June 1944, evacuations started once more when Karelia was reoccupied by the Soviet Union. This time the evacuations were more organised. This evacuation was final, and because returning to Karelia was not an option, the evacuees needed to settle permanently elsewhere in Finland and gradually integrate into society in the new locations (Waris, 1952). Overall, multiple Karelian parishes, cities and boroughs were lost partly or completely, and they are hereafter all called municipalities for clarity.

After the evacuations and the ending of the Continuation War, the Finnish government tried to help evacuees to settle into new municipalities in Finland, as well as reimburse a proportion of their lost possessions. The Finnish government-issued Land Acquisition Act in 1945 was created in order for war veterans, relatives of the fallen soldiers and evacuees to be able to get new land and homes after the war (Waris, 1952; Paukkunen, 1989). For Karelian evacuees, the placement plans were made as a part of the Act, which tried to place and settle farmers into Finland according to their original municipalities. The Act enabled Karelian farmers to receive land that had similar soil and climate to areas of their homes in Karelia, and it tried to keep old villages together in their designated areas as well. Thus, the placement plans supported the idea of keeping social groups together (Waris, 1952). However, the plans were not always followed perfectly, and in many cases people were moved around many times either by the government or by their own choice, and not always where the government planned to place them (Sinokki, 2019; Waris, 1952). Accordingly, the farmlands established were, on average, smaller and poorer in agricultural land than the original farms in Karelia (Paukkunen, 1989), thus people might have used any opportunity to purchase own land if it was possible, or move for other reasons.

### Data

The information about movements of the evacuees was collected into a book series called ‘Siirtokarjalaisten tie’ and this was afterwards digitised to create a life history database called Migrant Karelia (MiKARELIA) by Loehr et al. (2017). ‘Siirtokarjalaisten tie’ is a four volume book series about experiences of Karelian evacuees who lost their homes during the Winter War and Continuation War (Anonymous, 1970). Systematically recorded interviews, conducted by around 300 trained interviewers, took place between 1968 and 1970 to register entries for approximately 420,000 evacuees. For each person there is an entry that lists their full name (and possible maiden name when applicable), sex, date of birth, birthplace, profession, year of marriage, records of children (name, sex, birth date), record of membership in various organisations, and of all movements from birth to the date of the interview. If the person was married, the entry also lists the name, sex, date of birth, birthplace and occupation of their spouse. These books were scanned, and a software (Kaira Core and Natural Language Processing designed for use with the Finnish language) was developed to extract and digitise the records as a database called MiKARELIA (Loehr et al., 2017). Later additional information about family size and composition, individual's birth order, and brothers and sisters, has been added (Lynch et al., 2019b). This was done by extracting the information of siblings from records in a digitised database (Karjala-tietokanta database, n.d., [https://katiha.kansallisarkisto.fi/] recorded by the Finnish Lutheran and Orthodox Churches) and linking individuals between the databases by their name and exact day of birth. Each person has been given their own ID number. Individuals were also marked as the ‘primary person’ if they were the interviewed one of a married couple. For a more detailed description of how the data were extracted and constructed see Loehr et al. (2017) and Lynch et al. (2019b). Overall, the data consist of 250,000 individuals, including primary individuals, spouses and children. From this previously compiled database, we extracted data of individuals’ sex, year of birth, birth municipality, municipality in 1950, occupation, record of whether they returned to Karelia after the Winter War, number of brothers and sisters, and birth order (firstborns and laterborns). Our analysis focuses on a subset of individuals in this database who were over 18 years old in 1950, primary persons, farmers by occupation and with information available on their residency records, sex, year of birth and siblings.

The information about the placement plans has been gathered from a book called *Siirtokarjalaiset Nyky-Suomessa* (Paukkunen, 1989), which has small reports containing general overview of and information about evacuations and placement plans for all Karelian municipalities that were lost in the war. From these reports we extracted the placement municipalities in Finland for each Karelian municipality as instructed in the Land Acquisition Act of 1945 after the second evacuation. For example, for people from Hiitola, Karelia, the placement municipalities were Pori, Ulvila, Honkajoki, Karvia, Ahlainen, Merikarvia, Luvia, Nakkila, Siikainen, Kankaanpää, Kullaa, Pomarkku and Noormarkku in Finland according to the placement plans and the Land Acquisition Act (Paukkunen, 1989). While each Karelian municipality was assigned to multiple municipalities in the rest of Finland, smaller-scale communities (e.g. villages) were relocated to the same places.

By connecting the known records of an individual's current parish in 1950 and the placement plans, we were able to construct a variable for dispersal by 1950 for each evacuee in the dataset. Evacuees that lived in 1950 in one of the municipalities that had been assigned to their own Karelian municipality (birth municipality) in the placement plans would represent the people who stayed with their original in-group (dispersal = 0), and if they lived somewhere else, they had left their in-group (dispersal = 1). Those who had dispersed away had moved between the years 1944 and 1950. The year 1950 was chosen because the government-mandated settlement movement had ceased, and it was considered that the evacuees had found their permanent areas to settle by then (Waris, 1952). An individual's original birth municipality represented here the in-group because overall Karelians had their own culture, dialect, religion and other customs which often set them apart from the rest of Finns, and therefore it could be argued that Karelians felt more connected to their original community. These cultural differences might have even occasionally caused some conflict between Karelians and western Finns (Waris, 1952).

We focused only on the interviewed individuals in the original records, excluding individuals in the database that were spouses of the person interviewed because their information was incomplete (e.g. movements after evacuation are not listed) and they could not be considered as statistically independent observations from their partners. All the individuals that we focused on were farmers because the placement plans were made with them in mind, and therefore the analyses will be more accurate for the research, as the individuals form more cohesive groups. To be included in the analysis the subjects needed to be 18 years or older in 1950, as these individuals were old enough to move independently from their families. Data were also subset only to those who returned to Karelia after the lost Karelian areas were recaptured during the Continuation War and returning was possible. This is because the plans of the Land Acquisition Act, made alongside with the second evacuation plans, were more relevant to those that had to be evacuated again from Karelia in 1944 and who were under the pressure of finding a new home yet again. Those who did not return to Karelia after the regaining of the lost areas in the Continuation War might have already settled in non-occupied Finland following previous plans and therefore had not been as influenced by the newer placement plans. In other words, we focused on those who had to be evacuated the second time from Karelia in 1944.

### Statistics

All analyses were conducted with SAS Enterprise guide software version 8.2.1 (SAS Institute Inc., 2019), and graphs were done with R version 4.1.3 (R Core Team, 2022). Owing to limited information on all of the explanatory variables together, two separate models were conducted. Both analyses were done using generalised linear mixed models (GLIMMIX in SAS), with binomial distribution and logit link function. In both models dispersal (binary; 1 = individual had dispersed, and 0 = individual stayed) was the response variable, and birth municipality was set as a random factor. The explanatory variables were selected differently for each model; two models were conducted in order to have enough individuals of all ages in the analyses, as information about individuals’ number of siblings was not available for individuals younger than 27 years. The models are described below. Statistical significance is defined at the level of *α* = 0.05.

### Influence of sex and age on dispersal

We conducted one model to analyse the effects of sex and age on dispersal behaviour (*n* = 12,519). The explanatory variables for this model were sex (women *n* = 4342, men *n* = 8177), age as a continuous variable, and their interaction to test the hypothesis that age-specific differences in dispersal may be different for men and women. All individuals were between 18 and 60 years old (individuals over 60 years were considered as 60 years old because there were only a few people over the age of 60), and they were from 48 different Karelian municipalities (Supplementary Table S1A). We also ran the model with categorical age (5 year intervals) and the results remained the same. Altogether there were 6545 individuals who dispersed (2624 women, 3921 men) and 5974 individuals who stayed (1718 women, 4256 men). The aim is to test if men and women differ in dispersal behaviour to determine if dispersal is age related, and if there is a difference between men and women of different ages.

### Influence of brothers, sisters and birth order on dispersal

A second analysis focusing on siblings and birth order effects was conducted separately, owing to more limited data available for siblings (*n* = 4862). Here the explanatory variables were sex (women *n* = 1492, men *n* = 3370), age, number of brothers and sisters (both as continuous variables), whether or not an individual was firstborn (1 = yes, 0 = no), who are also referred to as firstborns and laterborns, and interaction between firstborn variable and number of brothers to test if the effects of brothers is different for those who are more likely, or not, to inherit the farm. A non-significant interaction between number of sisters and firstborn variable was removed. In the analysis of this subset, the most limiting factors were information of the number of brothers and sisters (missing for 61% of the individuals used in the first described model). The ages in this data subset varied from 27 to over 60 years old, because information about family size and composition was available only for individuals born before 1926. If an individual had more than five brothers or sisters, the variable value was recoded as 5 owing to the small sample size of firstborn individuals with more than five siblings (<10 individuals). In this subset individuals were from 41 different Karelian municipalities (Supplementary Table S1B). The aim is to understand if number of brothers or sisters influences the dispersal decisions of individuals; if firstborn and laterborn individuals behave differently; and to recognise if firstborns and laterborns behave differently regarding how many brothers and sisters they have.

## Results

### Influence of sex and age on dispersal

Descriptive statistics of the data (*n* = 12,519) show that on average women (*n* = 4342) had more dispersers (60.43%) than stayers (39.57%), whereas men (*n* = 8177) had on average more stayers (52.05%) than dispersers (47.95%). Descriptive statistics also show that the mean age of women who stayed with their in-group in 1950 was 41.6 years (dispersal = 0, SD = 12.50) and that of those who left their in-group was 39.9 years (dispersal = 1, SD = 12.38), whereas men who stayed were on average 39.5 years old (dispersal = 0, SD = 11.45) and those who dispersed were 40.4 years (dispersal = 1, SD = 11.32) in 1950.

**Figure 1.**
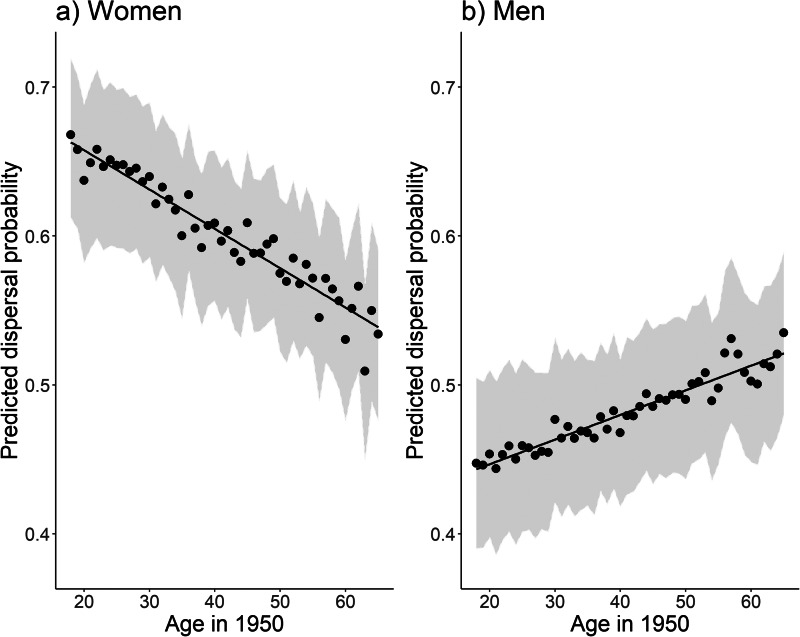
Black dots represent the mean of the model-predicted probabilities of dispersal for (a) women and (b) men by age in 1950. The black line is the predicted and grey areas are predicted confidence intervals for means. Predicted values were back-transformed to original scale.

The results of the analysis show that women had higher dispersal probability than men but the propensity to disperse was dependent on age (sex × age interaction: *F*_1,12515_ = 29.68, *p* < 0.0001). The younger the women were, the more likely it was that they dispersed, whereas men were more likely to stay with their in-group the younger they were. The model predicts that for women a one unit increase of age (e.g. from 30 to 31 years) decreased the odds of dispersal by 1.1% (OR = 0.989 [0.984–0.995], Figure 1), while for men these odds increased 0.7% (OR = 1.007 [1.003–1.011], Figure 1). For example, at the age of 25 the average predicted probability of dispersal for women is 29% higher than for men, but at the age of 50 the difference is only 15%.

### Influence of brothers, sisters and birth order on dispersal

Descriptive statistics of the sibling subset of the data (*n* = 4862) show that women had on average 2.7 brothers and 2.6 sisters, and men had 2.8 brothers and 2.7 sisters. There were 999 (women = 350, men = 649) firstborns and 3863 (women = 1142, men = 2721) laterborn individuals in the data.

**Figure 2.**
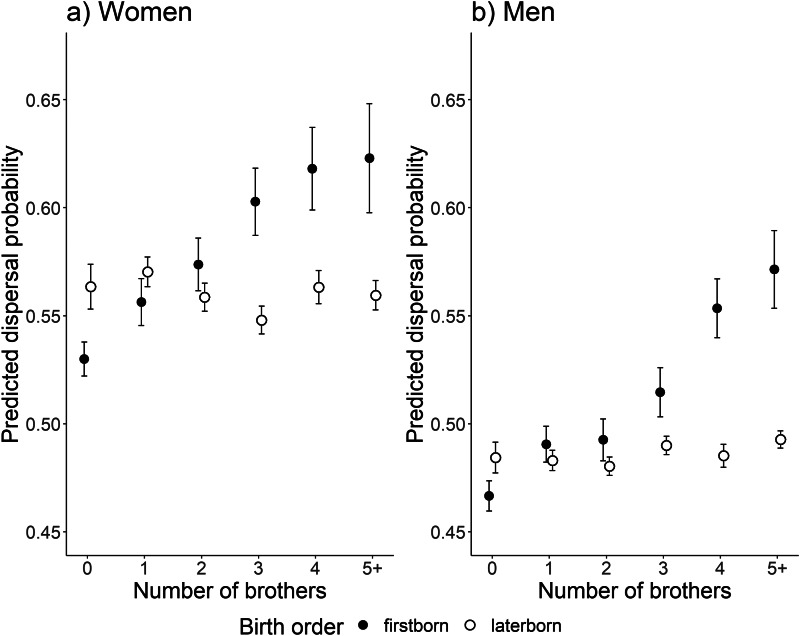
The filled dots represent model-predicted mean values of dispersal for firstborns and open dots for laterborns with error bars (standard error) by number of brothers, for both women and men. Brothers that are marked as ‘5+’ comprise a group of all individuals with five or more brothers. Predicted values were back-transformed to original scale. Men and women are represented as their own figures owing to their differing average predictions.

Results of the analysis also show that the effect of number of brothers depended on the birth order (firstborn or laterborn) (birth order × number of brothers interaction: *F*_1, 4854_ = 3.87, *p* = 0.0492). There was no significant effect of sex × birth order × number of brothers interaction (and this term was removed from the final analysis); therefore this effect of number of brothers for first- and laterborns was similar regardless of the focal individual sex. Similarly, the age × birth order × number of brothers interaction had no significant effect and was removed from the analysis. The model predicts that for firstborns a one unit increase in number of brothers (e.g. from 3 to 4) increases the odds of dispersing by 11% (OR = 1.110 [1.019, 1.209], Figure 2), while for laterborns the odds of dispersing increase only by 1.1% (OR = 1.011 [0.970,1.054], Figure 2). The number of sisters did not have a significant effect on dispersal probability (*F*_1,4854_ = 2.28, *p* = 0.1314, and neither did birth order × number of sisters interaction (removed from the model).

## Discussion

The resettlement of a large population of Karelians during World War II into new environments created a quasi-natural experiment to test how individuals’ dispersal behaviour might differ depending on their sex, age, birth order and sibling relations. We found support for the female-biased dispersal, though both men and women had dispersed away and stayed in their designated areas. However, even though there was a higher proportion of women dispersing than men, the difference was greatest at younger ages; while women dispersed mostly at younger ages, men stayed and were more likely to disperse when they were older. For women, the dispersal probability decreased with age and for men it increased and therefore the sex difference in dispersal probability evens out at older ages. This could be due to different pressures for men and women who are at the beginning of independence from parents, who are looking for spouses or work and have fewer established social connections, and most likely have less resources than older people.

Multiple previous studies of patriarchal societies have also found a greater proportion of women dispersing (Beise & Voland, 2008; Clarke & Low, 1992; Koenig, 1989; Towner, 2002), which indicates that there is a common trend that women are often the more dispersing sex in such contexts. Alternatively, some studies of matriarchal societies have found male-biased dispersal in humans, or no difference between dispersal tendencies between the sexes, indicating that the bias might be connected to societal structure (He et al., 2016). Female-biased dispersal is most common in patrilocal societies where males inherit land from their parents and women tend to follow marriages (Chen et al., 2023; Scelza, 2011; Marlowe, 2000; Koenig, 1989), and this similar dispersal sex bias is also usual with other mammals in which males defend resources, territories or partners (Greenwood, 1980). Men's resource gathering should also be a driving force for female-biased dispersal because then both sexes do not have to attain resources; women have been found to disperse more in association with marriage (Kok & Bras, 2008). Therefore, resource- and marriage-related dispersal could realistically explain some part of this female-biased dispersal of the younger individuals in the Karelian population, even though at this time we could not directly investigate this hypothesis with our data. In our study, young men's decisions to stay or leave might be more connected to resource availability and access than the decisions of young women. Historically in Finland, men owned land more than women, and (firstborn) sons inherited family farms more often than daughters (Faurie et al., 2009). Therefore, it makes sense that young men in this situation would be more inclined to grasp the opportunity of getting farming land when it is provided near a familiar social environment, possibly for support and help. Interestingly, a recent study done with the Karelians tested how local sex ratios were associated with the dispersal behaviour of women in urban and rural areas and found that women had higher probability of dispersing from less female-biased sex ratios, especially from rural areas compared with towns, and the authors concluded that the decisions of (single) women moving were not motivated by finding mates but rather by work in urban areas (Pettay et al., 2021). However, our data only focused on farmers, which could lead to differing interpretations between these studies, and therefore in this case we would not exclude the possibility of finding a spouse as a motivation for moving. Also, in our case, younger women might more easily integrate to new social environments and/or have greater overall pressure to find a spouse or work, while older women have already established their social groups and have spouses, therefore integrating into new societies might be less necessary. Findings of a previous study with the Karelians (Lynch et al., 2019b) are complementary to this assumption, where it was found that women were overall more likely to marry someone outside their in-group (non-Karelian Finns), and the same result is true for younger individuals of both sexes. Therefore, there could be a link between these two outcomes; if young women are most likely to disperse away from their in-group then it could, of course, result in overall greater intermarriage rates.

The benefits of staying with an in-group might have been greater – and the costs lower – for young men than for women in this study setting. It is also possible that the need for an in-group varies between ages and possibly that is why the dispersal probability was closer to 50% for both sexes for older individuals in 1950. This finding is contrary to standard patterns of dispersal of the older ages, where dispersing away from social groups is rarer. The high number of dispersing individuals among those who are over 50 years old might be due to the unique situation; everyone had to choose some place to live outside their original homes, and these individuals probably would not have moved in a normal situation. It is plausible that older individuals had more wealth when evacuating from Karelia, giving them increased opportunity to purchase larger farms outside their designated settlement areas. Decisions made at this juncture could be seen as a trade-off between staying with one's social group or having the opportunity for better land in a new area with a new social environment.

We found that the number of brothers had an effect on dispersal decisions for firstborn individuals of both sexes. Firstborns did not overall have a higher probability to leave than the laterborns but we found that the dispersal probability of firstborn individuals increases when the number of younger brothers increases. We found no indication that the dispersal probability is different within the laterborn individuals (Figure 2) as the number of brothers were greater. Interestingly, our finding was in contrast with our predictions and with previous discoveries where firstborn individuals possibly felt pressure from family to stay nearer their parents and younger siblings were more likely to disperse. A previous study with a historical Finnish population has found that especially eldest brothers who are inheritors of parent's property could increase the dispersal probability of their younger brothers in order to gain resources and avoid competition with close kin (Nitsch et al., 2016). The reason why our findings differ from this is most likely due to the different societal situations and contexts of the studies: historical pre-industrial agriculture era vs. our study in the post-war era of industrialisation and urbanisation. Furthermore, there have been mixed results for how siblings and birth order affect dispersal, which most likely depend on customs of the current time period and societies. In some studies, birth order has had no effect on the likelihood of dispersal (Beise, 2001; Towner, 2001), but other studies found linkage with these variables (Clarke & Low, 1992; Nitsch et al., 2016; Voland & Dunbar, 1997).

It is possible that in the unique situation of our study case – where the new farming lands were poorer in quality compared with those the farmers had in Karelia, and that the new lands were not old family farms that usually were inherited – the firstborns are not favoured anymore by their parents in the same way as they might have been favoured in Karelia, and instead might have felt more pressure to find a living elsewhere. This perhaps left more resources to younger brothers in the uncertain situation that the evacuees were in, and furthermore reduced competition with them. It is also possible that the firstborn individuals (perhaps only the younger ones) had better opportunities to leave their social group, especially if their younger brothers stayed near their families and offered to help their parents with their farms. However, to fully understand if inheritance practices of the families affected the dispersal in this situation more data on family dynamics, e.g. proximity of family members, before and after the evacuations would be needed.

Another contributing factor could be explained by the level of pre-migration wealth differences that the firstborns and laterborns possessed. Firstborn individuals could have already been favoured or helped by their parents in Karelia (for establishing their own farms and resources), and therefore they might have had the resources to search for better alternative farmlands elsewhere in Finland, similar to those who were older in age. If the firstborn individuals had better resources before the forced migration, and they had lived near their brothers in Karelia, it could be that them leaving the social group after the migration would leave bigger and better farming land in the designated areas for the remaining siblings. However, if only wealth was to explain this, then it raises the question why not all firstborns leave more rather than just those with many younger brothers. As there were many societal changes happening at the time of the study, it is possible that the dynamics of sibling interactions shifted as well. Therefore, more in-depth data from this and other populations is needed to better explain this surprising finding.

In contrast to brothers, sisters did not influence dispersal probability for either sex. Therefore, for firstborn women, a higher number of siblings of the opposite sex increased dispersal, while the same was true for firstborn men with same-sex siblings. Some studies have found sisters to promote staying (Beise & Voland, 2008), and sometimes elder sisters promote women's dispersal propensity (Nitsch et al., 2016). Perhaps, in this study the number of sisters does not influence dispersal because the sisters were anyway more likely to disperse away than brothers, or they were not as intensely influenced by the inheritance practices which creates less competition. However, sisters are not a pulling force for staying either, indicating there were no cooperative benefits from sisters.

In conclusion, we found that in mid-twentieth century Finland young women and men move differently under the pressure of finding a place to live, and firstborn individuals are influenced by their younger brothers to move away from their old social groups but not by their younger sisters. Our results challenge some general trends in the previous literature, calling for more research to focus on transitional societies such as the one after the war, where traditional migration patterns might be abandoned.

## Data Availability

The data that support the findings of this study are available in *Mendeley Data* at http://doi.org/10.17632/zm78bmt5tn.2

## Supplementary material

For supplementary material accompanying this paper visit https://doi.org/10.1017/ehs.2023.16. click here to view supplementary material
