# Supplementary material for: Sex, age, and family structure influence dispersal behaviour after a forced migration
Source: Evol Hum Sci. 2023 Jun 8;5:e21. doi: 10.1017/ehs.2023.16 (PMC10426002; doi:10.1017/ehs.2023.16)
Supplement: Supplementary file 1 [file S2513843X23000166sup001.docx]

Supplementary material to research article *Sex, age, and family structure influence dispersal away from social group after a forced migration.*

| **Supplementary Table S1.** List of all the birth municipalities (Karelia) of individuals in first model (A) and second model (B). |
| --- |
|  |
| 1. Antrea, Harlu, Heinjoki, Hiitola, Impilahti, Inkere, Jaakkima, Johannes, Jääski, Kanneljärvi, Kaukola, Kirvu, Kivennapa, Koivisto, Korpiselkä, Kuolemajärvi, Kurkijoki, Käkisalmi, Lavansaari, Lumivaara, Metsäpirtti, Muolaa, Pyhäjärvi Vpl., Pälkjärvi, Rautjärvi, Rautu, Ruskeala, Räisälä, Sakkola, Salmi, Seiskari, Soanlahti, Sortavala, Suistamo, Suojärvi, Suursaari, Säkkijärvi, Terijoki, Uukuniemi, Uusikirkko Vpl., Vahviala, Valkeasaari, Valkjärvi, Viipuri, Vuoksela, Vuoksenranta, Värtsilä, Äyräpää |
| 1. Antrea, Harlu, Heinjoki, Hiitola, Impilahti, Inkere, Jaakkima, Johannes, Jääski, Kanneljärvi, Kaukola, Kirvu, Kivennapa, Koivisto, Korpiselkä, Kuolemajärvi, Kurkijoki, Käkisalmi, Lumivaara, Metsäpirtti, Muolaa, Pyhäjärvi Vpl., Pälkjärvi, Rautjärvi, Rautu, Ruskeala, Räisälä, Sakkola, Soanlahti, Sortavala, Suistamo, Säkkijärvi, Terijoki, Uukuniemi, Uusikirkko Vpl., Vahviala, Valkjärvi, Viipuri, Vuoksela, Värtsilä, Äyräpää |
